# Facile biotic/abiotic sandwich detection system for the highly sensitive detection of human serum albumin and glycated albumin

**DOI:** 10.1007/s00216-024-05403-9

**Published:** 2024-07-15

**Authors:** Hirobumi Sunayama, Chehasan Cheubong, Eri Takano, Toshifumi Takeuchi

**Affiliations:** 1 Graduate School of Engineering, Kobe University, 1-1, Rokkodai-Cho, Nada-Ku, Kobe, 657-8501 Japan; 2 Department of Chemistry, Faculty of Science and Technology, Rajamangala University of Technology Thanyaburi, Klong Luang, Khlong Hok, 12110 Pathum Thani Thailand; 3 TearExo Inc., 1-1, Rokkodai-Cho, Nada-Ku, Kobe, 657-8501 Japan; 4 Innovation and Commercialization Division, Kobe University, 1-1, Rokkodai-Cho, Nada-Ku, Kobe, 657-8501 Japan; 5 Center for Advanced Medical Engineering Research & Development (CAMED), Kobe University, 1-5-1, Minatojima-minamimachi, Chuo-Ku, Kobe, 650-0047 Japan

**Keywords:** Abiotic/biotic sandwich detection system, Molecularly imprinted polymers, Post-imprinting modification, Diabetes, Glycated albumin

## Abstract

Quantifying glycated albumin (GA) levels in the blood is crucial for diagnosing diabetes because they strongly correlate with blood glucose concentration. In this study, a biotic/abiotic sandwich assay was developed for the facile, rapid, and susceptible detection of human serum albumin (HSA) and GA. The proposed sandwich detection system was assembled using a combination of two synthetic polymer receptors and natural antibodies. Molecularly imprinted polymer nanogels (MIP-NGs) for HSA (HSA-MIP-NGs) were used to mimic capture antibodies, whereas antibodies for HSA or GA were used as primary antibodies and fluorescent signaling MIP-NGs for the Fc domain of IgG (F-Fc-MIP-NGs) were used as a secondary antibody mimic to indicate the binding events. The HSA/anti-HSA/F-Fc-MIP-NGs complex, formed by incubating HSA and anti-HSA antibodies with F-Fc-MIP-NGs, was captured by HSA-MIP-NGs immobilized on the chips for fluorescence measurements. The analysis time was less than 30 min, and the limit of detection was 15 pM. After changing the anti-HSA to anti-GA (monoclonal antibody), the fluorescence response toward GA exceeded that of HSA, indicating successful GA detection using the proposed sandwich detection system. Therefore, the proposed system could change the detection property by changing a primary antibody, indicating that this system can be applied to various target proteins and, especially, be a powerful approach for facile and rapid analysis methods for proteins with structural similarity.

**Graphical Abstract:**

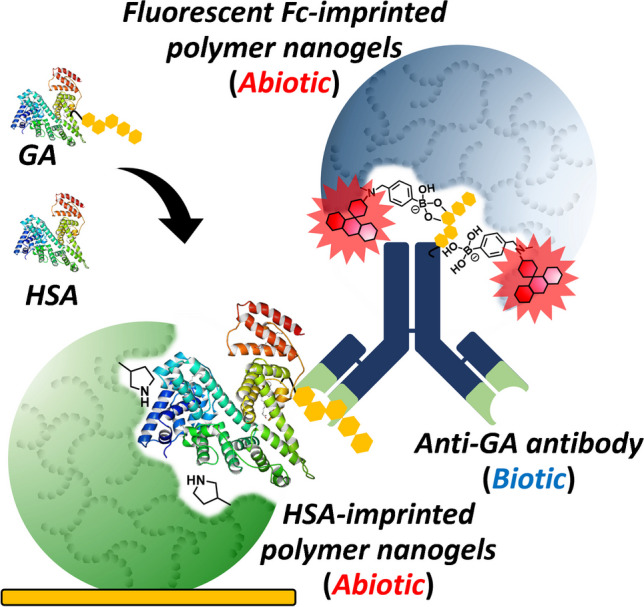

**Supplementary Information:**

The online version contains supplementary material available at 10.1007/s00216-024-05403-9.

## Introduction

The number of diabetes patients is increasing worldwide, estimated to be over 500 million [1]. The cost of medical care involving dialysis is rising with an increasing number of patients. In diabetes management, monitoring blood glucose levels is essential for understanding the effectiveness of the treatment. Commercially available glucose sensors incorporated with enzymes can measure patients’ blood glucose levels anytime. However, blood glucose levels frequently fluctuate throughout the day because of the intake of different meals and differing movements, necessitating periodic inspection. The invasive finger prick method for monitoring the blood glucose level by the sensor currently used imposes a significant burden on patients. To monitor the average blood glucose level within a given period, glycated hemoglobin (HbA1c) conjugated with sugar is used as a marker [2]. HbA1c half-life of ~ 30 days reflects the average blood glucose level within the past month [3]. It does not accurately reflect a patient’s glucose level most recent behavior, but rather that of a longer period. Similarly, glycated albumin (GA) is also used as a marker [4, 5], offering an attractive alternative for blood glucose indicator owing to its shorter half-life of ~ 17 days. GA reflects the average glucose level over a shorter period than HbA1c. During GA level monitoring, the ratio of GA to total human serum albumin (HSA) is referred for diagnosing diabetes. Therefore, it is important to develop facile and reliable methods for GA and total HSA detection.

High-performance liquid chromatography (HPLC) equipped with a boronic acid–functionalized column has been used for GA level measurements [6, 7]. Additionally, facile and precise analysis methods that use biomolecules, such as enzymes or antibodies, have been developed [8–11]. These biomolecule-based methods rely on the intrinsic molecular recognition abilities of the antibodies and enzymes. The sandwich assay method, known for its sensitive and selective detection, is frequently adopted. This method uses the capture antibodies immobilized on the substrate and primary antibodies for target molecule recognition and secondary antibodies labelled for transducing binding events, such as enzymes, fluorescent dyes, and radioisotopes. However, these strategies are time-consuming and expensive, and antibodies and enzymes are unstable under harsh conditions.

Molecularly imprinted polymers (MIPs) with molecular recognition abilities are promising alternatives to antibodies [12, 13]. MIPs are prepared by copolymerizing functional monomers with functional groups for interaction with target molecules, co-monomers, and cross-linking monomers in the presence of a target molecule. Subsequently, the target molecule is removed, resulting in a polymer matrix comprising a molecularly imprinted cavity with complementary size and shape to the target molecule, which is created and re-bound to the target molecule with high affinity and selectivity. MIPs are stable against chemical/physical stimuli because of their synthetic polymer-based nature. MIPs for various target molecules, including amino acids, sugars, herbicides, antibiotics, peptides, proteins, viruses, and bacteria, have been reported [14–16]. Various MIPs exhibit high affinity and selectivity comparable to those of natural antibodies. Additionally, MIP-based sensors have been prepared by combining various sensing systems, such as quartz crystal microbalances, surface plasmon resonance, colorimetry, fluorescence, amperometry, and voltammetry [17, 18]. An effective post-imprinting modification (PIM) strategy was developed to introduce other functionalities into the imprinted cavity [19, 20], where a functional monomer bearing a modifiable part, including a disulfide bond, an imine bond, and an amine group, was used for fabricating MIPs. Additionally, the functional group was introduced/transformed by chemically modifying the functional monomer residues within the cavity. PIM can selectively introduce a functional group into the cavity, inducing a low background signal and enhancing the signal/noise ratio [21, 22].

**Fig. 1:**
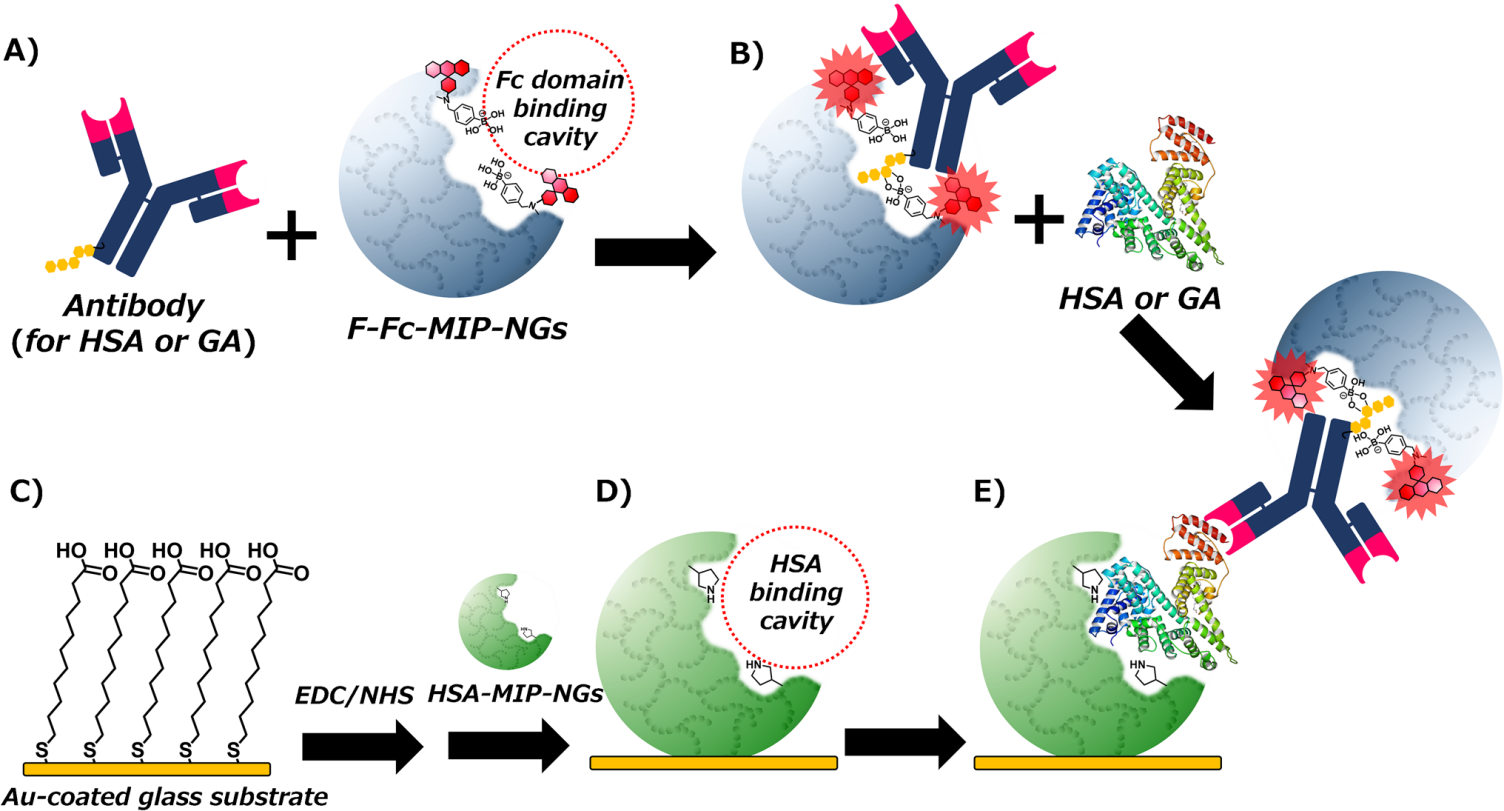
Schematic illustration of the biotic/abiotic sandwich detection system for human serum albumin (HSA) and glycated albumin (GA) detection. (A) Complex formation between antibody for HSA or GA and F-Fc-MIP-NGs; (B) mixture of the antibody-MIP-NG complex with target HSA or GA; (C) carboxy group–modified Au-coated sensor chip prepared via self-assembled monolayer formation; (D) HSA-MIP-NG immobilization on the sensor chip via EDC/NHS-mediated amine coupling; (E) biotic/abiotic sandwich detection using synthetic MIP-NGs and a natural antibody

Recently, a biotic/abiotic sandwich detection system combining MIPs and natural antibodies was developed and applied for Halal checks in meat extract samples [23]. This system combines the specificity of natural antibodies with the diverse functionalization flexibility of the synthetic polymer-based receptors. Food adulteration, specifically pork adulteration, is a significant concern for meat importers worldwide. Therefore, developing a facile and rapid method for detecting pork adulteration is critical. In a previous study, porcine serum albumin (PSA), selected as a pork adulteration marker protein, and PSA-imprinted polymer nanogels (PSA-MIP-NGs) were prepared and immobilized on the substrate [24]. Additionally, the Fc domain of human immunoglobulin G (IgG) imprinted polymer nanogels was also prepared using 4-(2-methacrylamidoethylaminomethyl) phenylboronic acid (MAPA) [21] as a modifiable functional monomer followed by the introduction of a fluorescent dye (ATTO647N) through PIM for fluorescent functionalization (F-Fc-MIP-NGs). The sandwich detection system was developed using PSA-MIP-NGs as a capture antibody mimic, an anti-PSA antibody as a primary antibody, and F-Fc-MIP-NGs as a secondary antibody mimic. Fluorescence measurements were conducted to study the sensing performance of the biotic/abiotic sandwich detection system for PSA detection. The analysis time for detecting pork adulteration of 0.01 wt.% in beef and lamb meat adulteration samples was less than 30 min. The detection limit achieved was comparable to those of commonly used immunoassays. In this sandwich detection system, which combines a natural antibody and functionalized synthetic polymer receptors, rapid, facile, and inexpensive detection of target proteins in complex meat extract samples was demonstrated. Only one antibody was used in this system, making it expected to establish a sensing system as an alternative to conventional ELISA, which is inexpensive to assemble and robust against chemical and physical stimuli. This study performs HSA and GA detection to expand the applicability of the aforementioned biotic/abiotic sandwich detection system (Fig. 1). HSA-imprinted polymer nanogels (HSA-NIP-NGs) [25], a polyclonal antibody for HSA, a monoclonal antibody for GA, and F-Fc-MIP-NGs were used for the proposed sandwich detection system assembly [23]. Additionally, fluorescence measurements were performed to investigate the sensing performance of the biotic/abiotic sandwich detection system for HSA and GA detection.

## Experimental

### Materials

Human immunoglobulin G (IgG), HSA, PSA, bovine serum albumin (BSA), fibrinogen (Fib), papain, 11-mercaptoundecanoic acid (MUA), *N*-hydroxysuccinimide (NHS), ethanolamine, sodium dodecyl sulfate, and DEAE–Sephadex A-50 were procured from Sigma-Aldrich (MO, USA). l-Cysteine, ethylenediamine tetra-acetate,* N*-isopropyl acrylamide (NIPAm), and *N*,*N′*-methylenebisacrylamide (MBAA) were purchased from Nacalai Tesque Co. (Kyoto, Japan). Sephadex G-100 was procured from GE Healthcare (Tokyo, Japan). Ethanol (EtOH), dimethyl sulfoxide, and 2,2′-azobis(2-methylpropionamidine) dihydrochloride (V-50) were procured from Wako Pure Chemical Industries, Ltd. (Osaka, Japan). 2-Methacryloyloxyethylphosphorylcholine (MPC) was purchased from the NOF Corporation (Tokyo, Japan). 1-(3-Dimethylaminopropyl)-3-ethylcarbodiimide hydrochloride (EDC) was purchased from Tokyo Chemical Industries (Tokyo, Japan). Methacryloxyethyl thiocarbamoyl rhodamine B was purchased from Thermo Fisher Scientific (MA, USA). The ATTO 647N NHS ester was purchased from ATTO-TEC GmbH (Siegen, Germany). The HiTrap™ Protein A HP column (1 mL) was procured from GE Healthcare, GmbH (Germany).

### Preparation of HSA-imprinted polymer nanogels (HSA-MIP-NGs)

**Scheme 1:**
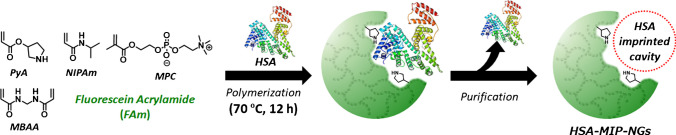
Schematic illustration of preparation of HSA-MIP-NGs

Pyrrolydin-3-ly acrylate (PyA) as a functional monomer for HSA imprinting was synthesized as previously reported [26]. Fluorescein acrylamide (FAm) as a landmark monomer was prepared using a previously reported procedure 25. HSA-MIP-NGs capable of HSA recognition were prepared using a previously reported procedure (Scheme 1) [25]. PyA was employed as a functional monomer capable of interaction with HSA. Briefly, HSA (13.2 mg), NIPAm (407 mg, 3.6 mmol), MBAA (30.8 mg, 0.2 mmol), MPC (59 mg, 0.2 mmol), PyA (42 mg, 0.3 mmol), FAm (4 mg, 0.01 mmol), and V-50 (217 mg) were dissolved in 10 mM phosphate buffer saline (PBS, pH 7.4, NaCl 140 mM) as a pre-polymerization solution. After removal of residual oxygen in pre-polymerization solution by a freeze-pump-thew method, emulsifier-free precipitation polymerization was carried out at 70 °C for 12 h. For purification and template removal, the obtained nanogels were passed through the ion-exchange chromatography (DEAE Sephadex A-50) and 10 mM PBS (pH 7.4) was used as an eluent. Fluorescence around 530 nm of each fraction (1.5 mL) was checked by fluorescent spectroscopy (*λ*_Ex_ = 491 nm, F-2500 fluorescence spectrophotometer, Hitachi High-Technologies, Tokyo, Japan). The particle size of the purified nanogels was measured by DLS measurement (Zetasizer Nano ZS, Malvern Instruments Ltd., Malvern, UK) and estimated to be 37 nm (PDI 0.735).

### Preparation of fluorescent signaling Fc domain–imprinted polymer nanogels (F-Fc-MIP-NGs)

First, template Fc domain was prepared by papain digestion of antibodies using the partially modified method as previously reported [23] with certain modification. Human IgG was digested with papain at 37 °C for 24 h. After that, the solution was filtered through an Amicon ultracentrifugal filter (10 kDa cutoff, 7500 × *g*, three times at 25 °C for 20 min) for desalting, followed by the buffer exchange with 20 mM phosphate buffer at pH 7.0. The obtained IgG fragments were first purified by ultrafiltration (100 kDa cutoff, 7500 × *g*, three times at 25 °C for 20 min) to separate the Fc domain (~ 50 kDa) from the whole IgG (~ 150 kDa). The collected solution was purified using HiTrap Protein A HP (1 mL).

4-(2-Methacrylamidoethylaminomethyl) phenylboronic acid (MAPA) as a functional monomer for Fc domain recognition was synthesized using a previously reported procedure [21].

The obtained Fc domain (2.5 mg, 50 nmol), MAPA (15.7 mg, 0.06 mmol), MTRB (0.42 mg, 0.63 μmol) as a fluorescent monomer, MPC (3.7 mg, 0.012 mmol) and NIPAm (102 mg, 0.9 mmol), MBAA (7.71 mg, 0.05 mmol), and V-50 (54.2 mg, 0.2 mmol) were dissolved in 10 mM carbonate buffer containing 2% dimethyl sulfoxide (pH 9.2, 25 mL). Then, the polymerization was initiated thermally and stirred at 50 °C for 12 h. After polymerization, the solvent was exchanged with PBS using ultrafiltration with 10 kDa cutoff (7500 × *g*, three times at 25 °C for 20 min), and the collected nanogels were incubated with SDS aqueous solution (40 mg/mL, 1 mL) for 5 min at 25 °C. The template (Fc domain) removal was performed with size-exclusion chromatography followed by anion-exchange chromatography, as reported previously [23]. Finally, further purification to remove SDS was performed using a PD-10 column (Desalt column) with elution by PBS (3.5 mL) for removing SDS. To collect the nanogel fractions, fluorescence from the MTRB residues was used as a marker at *λ*_em_ of 575 nm (*λ*_ex_ 548 nm).

F-Fc-MIP-NGs were prepared via post-imprinting modification (PIM) with ATTO 647N NHS ester as the fluorescence reporter dye. The obtained Fc-MIP-NGs (500 μg/mL, 1000 μL) were incubated with 5 μL of 10 mg/mL ATTO 647N NHS ester in DMSO at 25 °C for 2 h. The unreacted fluorescent dye was then removed using an Amicon ultracentrifugal filter (10 kDa cutoff, 7500 × *g*, three times at 25 °C for 20 min) with PBS.

### Immobilization of HSA-MIP-NGs on the sensor chip

The sensor chip (gold-coated glass substrate; 4.3 × 9.8 mm, Au 165 nm thickness and Ti 5 nm thickness) was washed with pure water and EtOH followed by UV-O_3_ treatment for 20 min. The cleaned sensor chip was immersed in an ethanolic solution containing MUA (1.0 mM) for 24 h at 25 °C for surface modification by thiol-Au interaction–based self-assembled monolayer (SAM) formation. After washing the sensor chip with pure water and EtOH, 100 µL of an aqueous solution containing 0.2 M EDC and 0.05 M NHS was dropped onto the sensor chip and incubated for 2 h at 25 °C. After washing with pure water, 100 µL of 10 mM phosphate buffer saline (PBS, 140 mM NaCl, pH 7.4) containing HSA-MIP-NGs (100 µg/mL) was dropped onto the sensor chip and incubated for 1 h at 25 °C. After immobilizing MIP-NGs, a 1 M ethanolamine aqueous solution (pH 8.5, 100 μL) was added to block the unreacted NHS ester, and the reaction proceeded for 30 min at 25 °C. Finally, PBS (100 µL) containing 0.5 wt.% BSA was added as a blocking agent to suppress non-specific binding on the sensor surface.

For confirming the immobilization of the HSA-MIP-NGs on the sensor chip, the fluorescence of the HSA-MIP-NGs immobilized sensor chip was measured by fluorescent microscope with custom-made liquid handling robot (System Instruments Co. Ltd., Tokyo, Japan) [27, 28]. The sensor chip was put into the pipette chip with flat part for fluorescent measurements and set on the robot arm. Then, PBS (150 µL) was aspirated and moved to the detection port. Fluorescent microscope conditions were as follows; Zyla 5.5 sCMOS camera (Andor Technology Ltd, Belfast, UK) equipped with a fluorescence turret (BX3-URA, Olympus, Tokyo, Japan); 5 × objective lens (LMPLFLN5X, Olympus, Tokyo, Japan); exposure time, 0.1 s; light source, mercury lamp (HGLGPS-SET, Olympus, Tokyo, Japan); bandpass filters (BWA: 460–495 nm for excitation, 510–550 nm for emission, and DM 505 nm).

Fluorescence intensities of an initial surface (*F*_0_ = *F*_only substrate_) on a sensor chip and fluorescence intensity changes after HSA-MIP-NGs immobilization (*F* − *F*_0_ = *F*_immobilized NGs_–*F*_only substrate_) were used for calculation of the relative fluorescent intensity by the equation, (*F* − *F*_0_)/*F*_0_.

### Biotic/abiotic sandwich detection system

The cocktail solution was prepared by mixing equal volumes of PBS containing 0.1 µg/mL anti-HSA (polyclonal or monoclonal antibody) with F-Fc-MIP-NGs (100 μg/mL) followed by incubation for 30 min. Varying concentrations of HSA (0–100 nM) were added to the above reaction mixture. Subsequently, the premixed cocktail solution was dropped onto the HSA-MIP-NG-immobilized sensor chip and incubated for 30 min. After washing with pure water (3 × 500 μL) and PBS (3 × 500 μL), the sensor chip was placed into a flat-type pipette tip, followed by aspiration of 150 μL PBS. The fluorescence intensity was measured using a custom-made liquid handling robot equipped with a fluorescence microscope (System Instruments Co., Ltd. Tokyo, Japan) [27, 28] under the sequence described below. First, the flat-type pipette tips were placed on the tip rack, which was captured using a robot arm. Next, PBS (150 μL) was aspirated, and the robot arm was moved to the detection port to capture a surface image of the sensor chip. Subsequently, the fluorescence intensity was measured using a Zyla 5.5 sCMOS camera (Andor Technology Ltd, Belfast, UK) equipped with a fluorescence turret (BX3-URA, Olympus, Tokyo, Japan). The experiments were performed in triplicate, and six different regions of interest (ROIs) were selected from each chip (5 × objective lens; LMPLFLN5X, Olympus, Tokyo, Japan). The measurement conditions include exposure time, 0.1 s; light source, mercury lamp (HGLGPS-SET, Olympus, Tokyo, Japan); bandpass filters (Cy5); 604–644 nm for excitation; and 672–712 nm for emission. The relative fluorescence intensity of the binding experiments was calculated using the equation (*F* − *F*_0_)/*F*_0_, where *F*_0_ and *F* are the fluorescence intensities before and after incubation, respectively. Ten nanomolars of reference proteins (PSA, IgG, and Fib) dissolved in PBS was added to the cocktail solution to investigate the selectivity of the developed sensor for HSA.

The above procedure utilized anti-GA monoclonal antibodies to prepare the cocktail solution for GA detection instead of anti-HSA antibodies. Fluorescent responses of the sensor chip with addition of various concentrations of GA or HSA (0–100 nM) were measured.

## Results and discussion

HSA-MIP-NGs were prepared via emulsifier-free precipitation polymerization, as previously reported [25]. Hydrophilic and biocompatible NIPAm and MPC [29] were the primary components of the polymer; pyrrolidne-3-yl acrylate was used as a functional monomer [26], MBAA as a cross-linking monomer, and fluorescein acrylamide as a landmark monomer (Scheme 1). Following MIP-NG immobilization on the sensor chip surface, the fluorescence intensity from the fluorescein incorporated in the polymer matrix increased, indicating successful immobilization of HSA-MIP-NGs on the sensor chip surface (Figure S1).

**Scheme 2:**
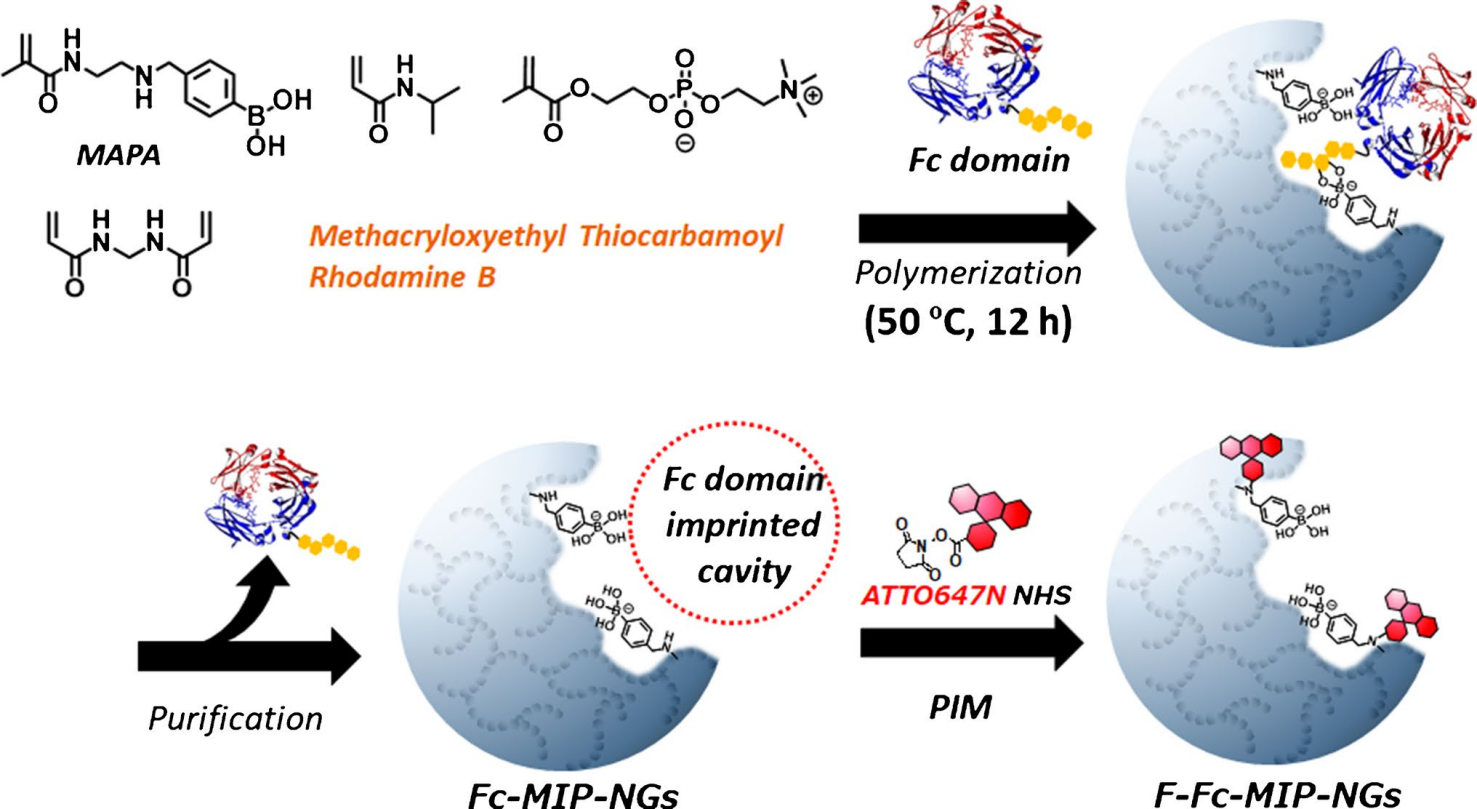
Schematic illustration of preparation of F-Fc-MIP-NGs

The F-Fc-MIP-NGs were prepared based on a previously reported procedure [22]. Fc domain-imprinted polymer nanogels were also prepared via emulsifier-free precipitation polymerization, using NIPAm and MPC as the main monomers and MAPA as a functional monomer that interacts with sugar chains via cyclic di-ester formation and with amino acid residues via electrostatic interaction (Scheme 2). After purification of NGs and template Fc domain removal, ATTO647N reacted with the amine group on the MAPA residue within the imprinted cavity to introduce a fluorescent signaling function (F-Fc-MIP-NGs). F-Fc-MIP-NGs capture the Fc domain of IgG exhibiting high affinity (*K*_d_ ≈ 10^−8^ M) and selectivity and transduce the binding events to fluorescent changes.

**Fig. 2:**
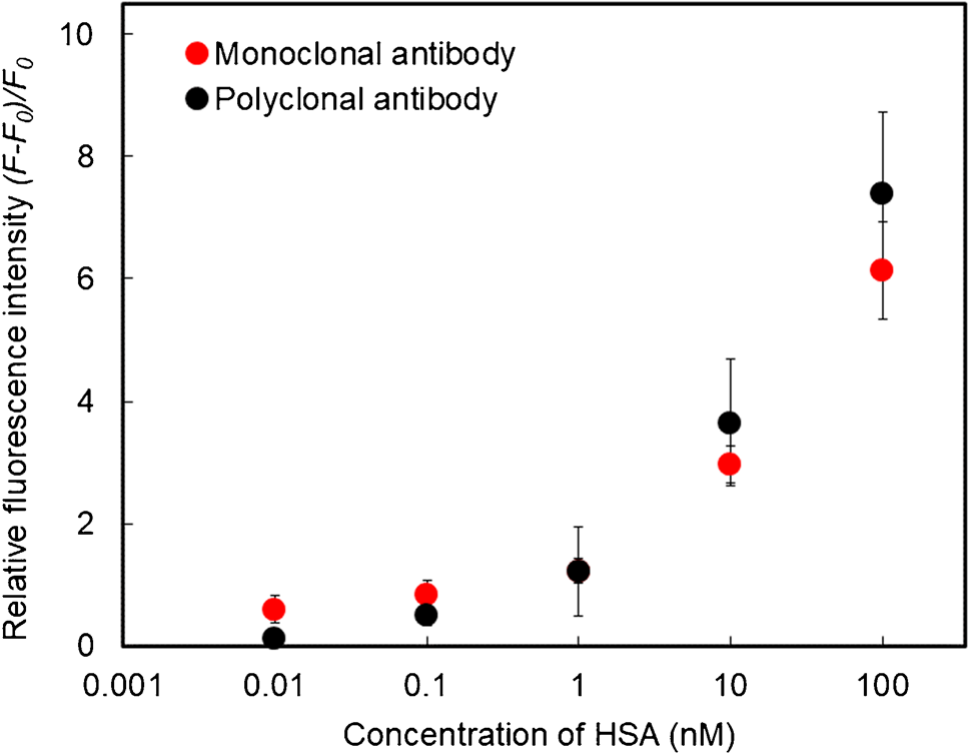
Fluorescent responses of the developed biotic/abiotic sandwich detection system to HSA (0–100 nM), with polyclonal antibody for HSA (black) and monoclonal antibody for HSA (red) (*n* = 3)

A biotic/abiotic sandwich detection system was performed for HSA detection using an HSA-MIP-NG-immobilized sensor chip, an anti-HSA antibody, and F-Fc-MIP-NGs. The assay conditions were optimized based on a previous study, using 0.5 wt.% BSA as the blocking reagent, 0.1 µg/mL of antibody, 100 µg/mL of F-Fc-MIP-NGs, and a 30-min reaction time [23]. The HSA-sensing ability of the proposed sandwich detection system using polyclonal and monoclonal antibodies was examined using fluorescence microscopy (Fig. 2). This fluorescent microscope was equipped with a custom-made liquid handling robot, enabling the control of liquid handling steps involving aspiration of the sample solution, incubation at the incubation port, and transfer to the detection port by computer [27, 28]. Therefore, automating these steps enables facile and rapid procedures for fluorescent measurement. The fluorescent intensities of both assays increased with HSA addition, demonstrating the effective detection of HSA by the proposed detection system. The limit of detection values were estimated using the 3SD/*m* equation (where SD is the standard deviation for 0 nM HSA and *m* is the slope of the linear area of the binding isotherms). The calculated values were 23 and 15 pM for the monoclonal and polyclonal antibodies, respectively (Figure S2). Generally, the concentration of HSA in blood is 35–50 mg/mL (0.5–0.75 mM), and the GA level (the ratio of GA to total albumin) in healthy individuals is 11–16%. In patients with diabetes, the GA level can rise to over 20%. Therefore, the proposed detection system shows the potential to detect HSA and GA even in serum diluted 10,000-fold. These values show higher sensitivity than those obtained using previously reported MIP-based sensors and are comparable to those obtained using commercially available ELISA kits [30, 31]. In the low-concentration area of the binding isotherms, the fluorescent response of the detection system using a monoclonal antibody for HSA was more significant than that using a polyclonal antibody, implying that the epitopes of polyclonal antibody and recognition moieties of HSA by capture HSA-MIP-NGs are competitive. The monoclonal antibody used in this study was unlikely to compete with the binding domain of HSA for HSA-MIP-NGs. Conversely, in the high-concentration area of the binding isotherms, the fluorescent response of the detection system using the polyclonal antibody was more significant than that obtained using the monoclonal antibody, indicating that the existing amount of HSA was sufficient to suppress the competition between the binding domain and HSA-MIP-NGs.

**Fig. 3:**
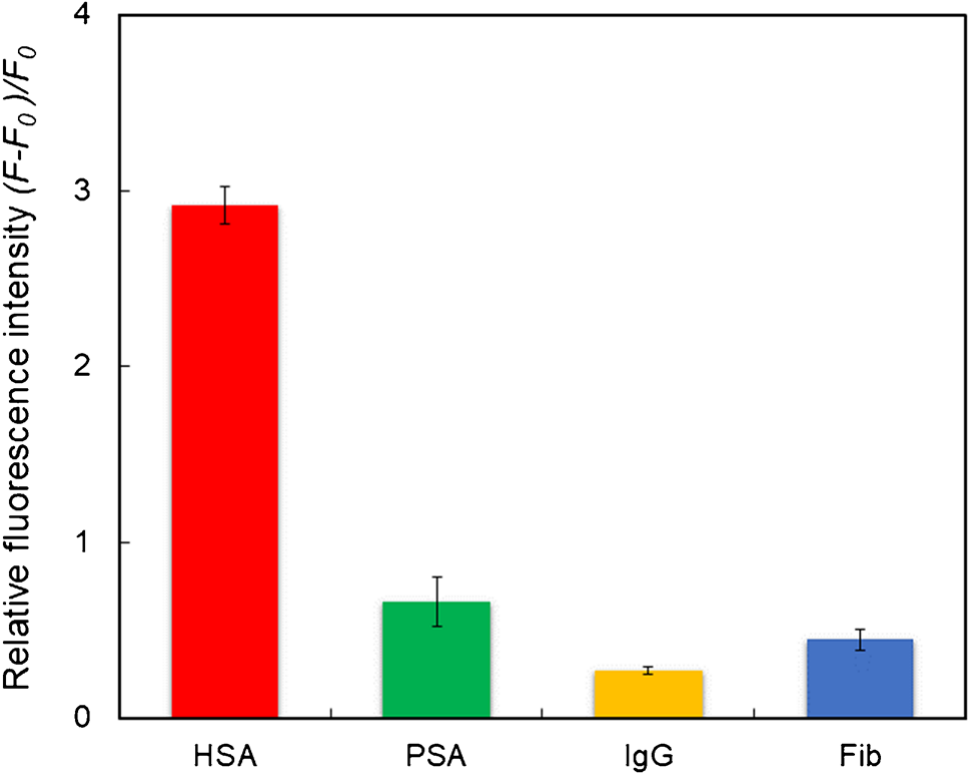
Relative fluorescence intensities of the proposed sandwich detection system for HSA and reference proteins: porcine serum albumin (PSA), immune globulin G (IgG), and fibrinogen (Fib). The protein concentrations were 10 nM. A fluorescence microscope with a Cy5 filter (*λ*_ex_ 604–644 nm and *λ*_em_ 672–712 nm) was used (*n* = 3)

To examine the selectivity of the proposed biotic/abiotic detection system, IgG, Fib, and PSA were used as reference proteins at a concentration of 10 nM each. IgG and Fib are popular plasma proteins, and PSA is albumin derived from pigs (Fig. 3). The fluorescence response of the detection system toward HSA was greater than that of the reference proteins, confirming the selectivity of the detection system for HSA detection. Notably, this detection system exhibited higher selectivity for HSA detection than our previously reported single MIP-based sensors [26, 30], suggesting that the combination of biotic antibodies and abiotic MIP-NGs enhanced the affinity and selectivity of the developed system for capturing and detecting HSA in solution. Furthermore, it has been demonstrated that MIP-NG-based materials retain their original molecular recognition ability or specific sensing ability when applied in biological fluids, such as blood and meat extracts [25, 30, 32, 33]. These findings imply that the proposed sandwich detection system could also be effective with biological samples.

**Fig. 4:**
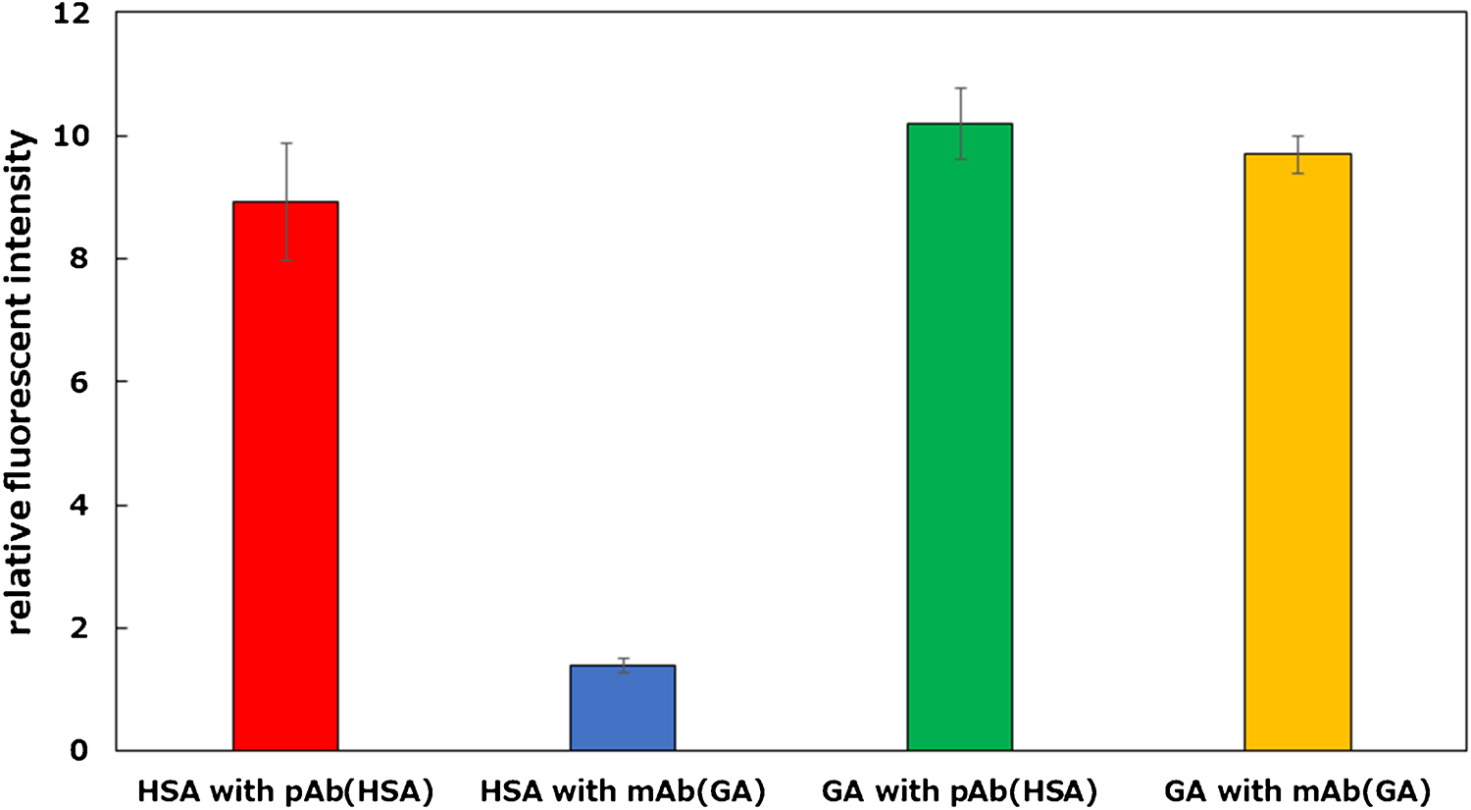
Fluorescent responses toward HSA and GA (100 nM) of the developed biotic/abiotic sandwich detection system using the polyclonal antibody for HSA (pAb (HSA)) or the monoclonal antibody for GA (mAb (GA))

To detect GA, a biotic/abiotic sandwich detection system was performed using a monoclonal antibody against GA instead of an antibody against HSA. The fluorescent responses increased upon adding GA (0.01–100 nM); however, those toward HSA did not increase (Figure S3). These results indicate that the developed sandwich detection system could selectively and sensitively detect GA. Figure 4 shows the fluorescent responses of the developed sandwich assay toward HSA or GA (100 nM each) using a polyclonal antibody for HSA or a monoclonal antibody for GA. As described above, a system using a monoclonal antibody for GA can selectively detect GA. In contrast, the fluorescent responses for HSA and GA were nearly equivalent in the detection system using a polyclonal antibody for HSA, indicating that this system could be used to quantify total albumin and GA. Therefore, by simply changing the antibody, the proposed biotic/abiotic sandwich detection system could rapidly measure total albumin and GA (~ 30 min) using facile procedures. These performances were comparable to recently reported sensing systems, such as paper-based immunosensors and electrochemical sensors. Furthermore, the sensitivity of the developed sensing system was also similar to that of commercially available ELISA kits (Table S1). This method implies that the proposed system could rapidly analyze proteins with similar structures by changing the antibody or MIP-NGs to capture the target protein.

## Conclusion

Biotic/abiotic sandwich detection systems were developed using HSA-MIP-NGs, antibodies, and fluorescent signalling F-Fc-MIP-NGs to detect the target binding. The abiotic components comprise MIP-NGs for HSA and the Fc domain, whereas the biotic components include natural antibodies for HSA and GA. The proposed sandwich assay could specifically detect HSA and GA in the picomolar order by changing only the antibodies. Combining the natural antibodies with the synthetic receptors prepared through molecular imprinting followed by post-imprinting modifications enables the construction of a facile, easy, and rapid (< 30 min) sandwich detection system, potentially evaluating GA levels for monitoring the blood glucose level. Various MIP-based functional materials can be tailored using different template molecules and adapting PIMs. Additionally, corresponding antibodies for various targets are commercially available. Therefore, this easy and specific sensing system would be a powerful tool for analyzing proteins in life sciences fields, such as diagnosis, therapeutics, food, and environmental analyses.

## Supplementary Information

Below is the link to the electronic supplementary material.

Supplementary file1 (PDF 780 KB)
