# Supplementary material for: Facile biotic/abiotic sandwich detection system for the highly sensitive detection of human serum albumin and glycated albumin
Source: Anal Bioanal Chem. 2024 Jul 15;416(30):7337–45. doi: 10.1007/s00216-024-05403-9 (PMC11584489; doi:10.1007/s00216-024-05403-9)
Supplement: Supplementary file 1 — Supplementary file1 (PDF 780 KB) [file 216_2024_5403_MOESM1_ESM.pdf]

**Supporting Information for**

# *Facile Biotic/Abiotic Sandwich Detection System for the Highly Sensitive Detection of Human Serum Albumin and Glycated Albumin*

*Hirobumi Sunayama [1]*, Chehasan Cheubong [1,2], Eri Takano [3], Toshifumi Takeuchi [4,5]**

[1] Graduate School of Engineering, Kobe University, 1-1, Rokkodai-cho, Nada-ku, Kobe 657-8501, Japan

[2] Department of Chemistry, Faculty of Science and Technology, Rajamangala University of Technology Thanyaburi, Pathumthani 12110, Thailand

[3] TearExo, Inc., 1-1, Rokkodai-cho, Nada-ku, Kobe 657-8501, Japan

[4] Innovation and Commercialization Division, Kobe University, 1-1, Rokkodai-cho, Nada-ku, Kobe 657-8501, Japan

[5] Center for Advanced Medical Engineering Research & Development (CAMED), Kobe University, Chuo-ku, Kobe 650-0047, Japan

* Corresponding author: Toshifumi Takeuchi, Center for Advanced Medical Engineering Research & Development (CAMED), Kobe University, Chuo-ku, Kobe 650-0047, Japan, TEL/FAX: +81 78 803 6158, E-mail address: takeuchi@gold.kobe-u.ac.jp; Hirobumi Sunayama, Graduate School of Engineering, Kobe University, 1-1, Rokkodai-cho, Nada-ku, Kobe 657-8501, Japan, Email address: sunayama@penguin.kobe-u.ac.jp

# Contents

**1. Fluorescence measurements of HSA-MIP-NGs immobilized sensor chip**........................... 3
**2. Calibration curves for HSA** ........................................................................................... 4
**3. Fluorescence detection by the developed sandwich assay for GA and HSA** ....................... 5
**4. Comparison of developed sensing system with previously reported HSA and GA sensors**6

## 1. Fluorescence measurements of HSA-MIP-NGs immobilized sensor chip

The fluorescence of the HSA-MIP-NGs immobilized sensor chip was measured by fluorescent microscope with custom-made liquid handling robot (System Instruments Co. Ltd., Tokyo, Japan).[1]

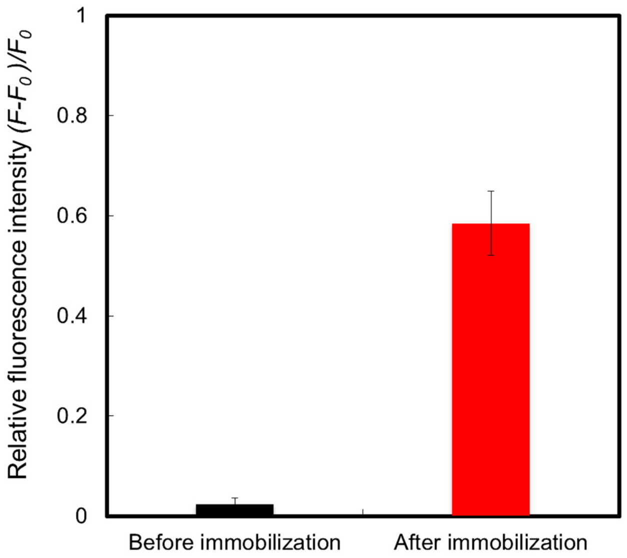


**Figure S1.** Relative fluorescent intensities of the sensor chips, substrate only and after HSA-MIP-NGs immobilization.

## 2. Calibration curves for HSA

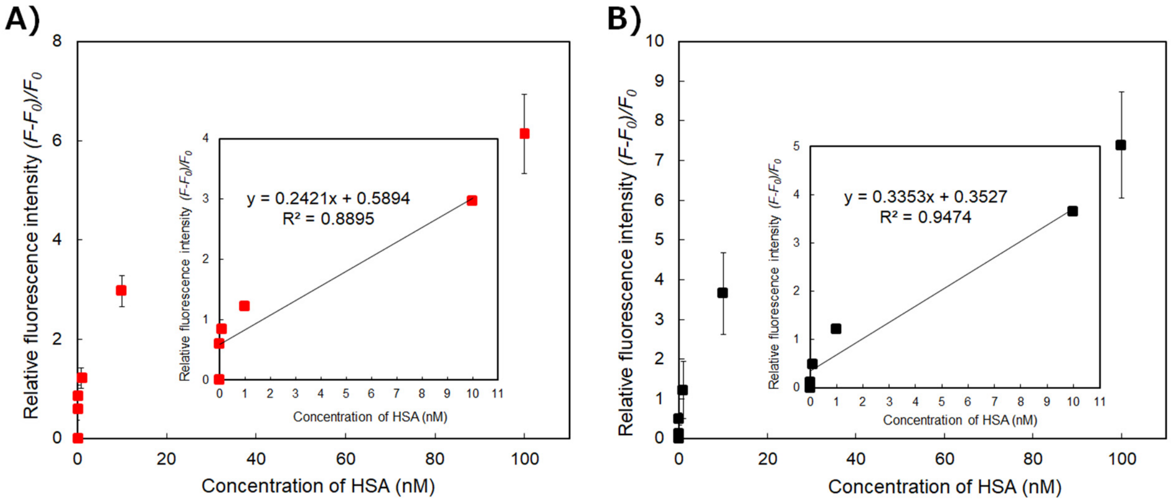


**Figure S2.** The calibration curves of the developed sandwich assay for HSA (0-100 nM) shows a linear calibration range of 0.01–10 nM ($r^2$ = 0.8895 for monoclonal antibody and 0.9474 for polyclonal antibody). The error bars were obtained from triplicate experiments.

### 3. Fluorescence detection by the developed sandwich assay for GA and HSA

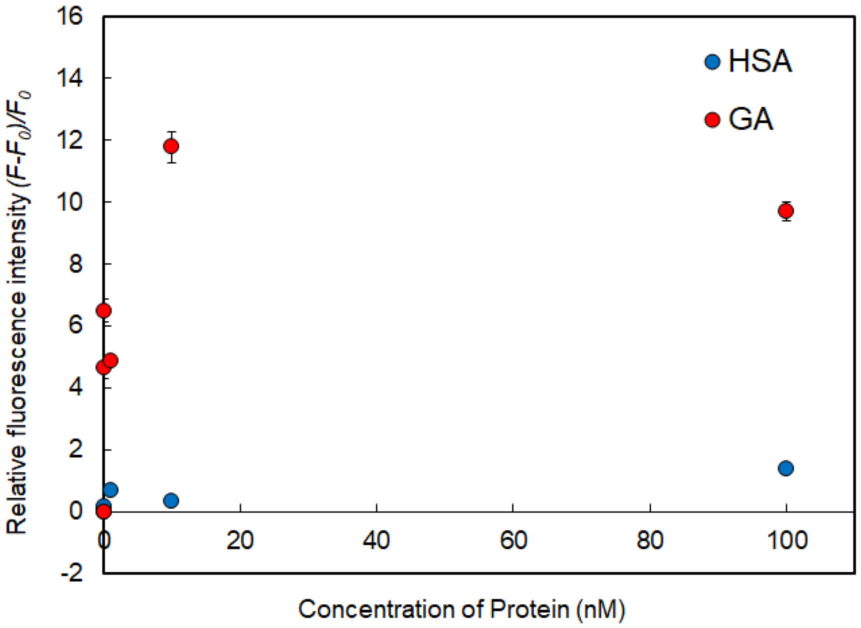


**Figure S3.** Fluorescent responses towards GA or HSA of developed biotic/abiotic sandwich assay using monoclonal antibody for GA.

## 4. Comparison of developed sensing system with previously reported HSA and GA sensors

**Table S1.** Comparison of the proposed sensing system with previously reported sensors towards HSA and/or GA.

| Sensor platform | Molecular recognition element | HSA detection range | GA detection range | Detection limit | Detection time | Ref No. |
|---|---|---|---|---|---|---|
| Label-free impedimetric immunosensor | Antibody (x1) | 10 ng/mL, 50 ng/mL | 7.49%-22.56% (10 ng/mL) 7.49%-15.79% (50 ng/mL) | - | ~20 min | [2] |
| Enzymatic electrochemiluminesence | - | - | 0.1 -10 μM | 0.1 μM | > 1 h | [3] |
| Boronic acid agarose bead lateral flow assay | Bromocresol green, Boronic acid-modified agarose beads | 10 – 50 mg/mL | 0.01 – 10 mg/mL | 7.1 μg/mL | ~ 20 min | [4] |
| Paper-based immunoassay | Antibody (x2) | 1 ng/mL – 1 mg/mL | 0.5 μg/mL – 3.6 mg/mL | 49.16 ng/mL 8.36 μg/mL | - | [5] |
| Aptamer-based electrochemical sensor | Aptamer, antibody | 0.1, 1, 10 mg/mL | 0.1, 1, 10 mg/mL | - | >30 min | [6] |
| Sandwich ELISA (Commercially available) | Antibody (x2) | - | 1.563 – 100 nM | 0.938 nM | > 3 h | [7] |
| Biotic/abiotic sandwich detection system | MIP-NGs, antibody | 0.01 – 100 nM | 0.01 – 100 nM | 23 pM | ~30 min | This work |

### References


[1] E. Takano, N. Shimura, Y. Ujima, H. Sunayama, Y. Kitayama, T. Takeuchi, T. *ACS Omega* **2019**, 4, 1487-1493.

[2] N. Bohli, O. Meilhac, P. Rondeau, S. Gueffrache, L. Mora, A. Abdelghani, *Talanta*, **2018**, 184, 507–512.

[3] Y. Inoue, M. Inoue, M. Saito, H. Yoshikawa, E. Tamiya, *Anal.. Chem.* **2017**, 89, 5909−5915.

[4] E. Ko, V. K. Tran, Y. Geng, M. K. Kim, G. H. Jin, S. E. Son, W. Hur, G. H. Seong, *Biomicrofluidics,* **2018**, 12, 014111

[5] H. Ki, H. Jang, J. Oh, G-R. Han, H. Lee, S. Kim, M-G. Kim, *Anal. Chem*., **2020**, 92 (17), 11530-11534

[6] T. Sakata, R. Shiratori, S. Nishitani, *Anal. Chem*., **2023**, 95 (2), 1480-1489.

[7] antibodies-online.com, https://pdf.antibodies-online.com/productsheets/ABIN6967133.pdf (access date; 23. Apr. 2024)
